# A computer study of the risk of cholesterol gallstone associated with obesity and normal weight

**DOI:** 10.1038/s41598-021-88249-w

**Published:** 2021-04-23

**Authors:** Krystian Kubica, Joanna Balbus

**Affiliations:** 1 Faculty of Fundamental Problems of Technology, Department of Biomedical Engineering, Wroclaw University of Science and Technology, 50-370 Wroclaw, Poland; 2 Department of Pure and Applied Mathematics, Wroclaw University of Science and Technology, 50-372 Wroclaw, Poland

**Keywords:** Computational biology and bioinformatics, Risk factors

## Abstract

Obese people differ from the people of normal weight in gall bladder motility and have a higher risk of cholesterol stone formation. In this study, using a mathematical model of cholesterol homeostasis, which also considers the enterohepatic circulation of bile as well as cholesterol, we investigated the risk of cholesterol stone formation in obese and normal-weight groups who had normal blood cholesterol levels. We associated the risk of stone formation with the amount of cholesterol released into bile and the amount of de novo-synthesized cholic acid. For both groups, we determined the conditions of low and high risk. In addition, we analyzed the potential effects of changes in gall bladder motility with increased weight. The results showed that the obese group exhibited increased kinetics of enterohepatic circulation, leading to a significant increase in blood cholesterol levels, which can be reduced by increasing the amount of cholesterol in bile. Based on this finding, we suggest that for obese people, it is beneficial to reduce the amount and change the composition of circulating bile through the inhibition of cholic acid synthesis along with cholesterol synthesis. Furthermore, obese people should maintain a triglyceride-lowering diet and consume small meals containing fat, preferably in combination with agents that can reduce bile output from the gall bladder.

## Introduction

Wrong diet and low physical activity, together with genetic predisposition and diseases, are the causes for the growing number of overweight and obese people. The World Health Organization has reported that 39% of adults (age > 18 years) (40% women and 39% men) are overweight (body mass index (BMI) > 25 kg/m^2^) and 13% of adults (15% women and 11% men) are obese (BMI > 30 kg/m^2^) (www.who.int/news-room/fact-sheets/detail/obesity-and-overweight).

Obesity is associated with many diseases and anatomical and physiological conditions: greater strain on the joints and a greater risk of hypertension, type II diabetes, night apnea, cholelithiasis, and blood lipid disorders. In addition, in the obese group, a higher serum cholesterol level is observed, although the literature data show a high variability^1,2^. Changes in gall bladder motility are commonly observed with obesity^3^. Obese people usually exhibit a larger gall bladder fasting volume—up to 33.0 ± 2.0 mL^4^. Moreover, the gall bladder residual volume in these people is significantly higher compared to normal-weight people (obese: 8.0 ± 0.8 mL, normal weight: 5.8 ± 0.3 mL)^4,5^. In lean people, the gall bladder eliminates about 75% of its content after a meal^4^. The parameters describing gall bladder motility in obese patients depend on BMI, age, and other individual features^6,7^ and slightly differ according to the research^3,4,6,8^. In the present work, we refer to the data reported in the study of Di Ciaula et al.^4^. According to these authors, the gall bladder emptying pattern significantly differs between normal-weight and obese subjects. In obese people, the emptying rate is about 0.46 mL/min, whereas in normal-weight people, it is only about 0.3 mL/min^4^. However, the duration of gall bladder emptying is similar in both groups, i.e., approximately 45 min^4^.

Furthermore, between obese and normal-weight people, a large difference has been observed in the duration of gall bladder refilling, wherein 75% of fasting volume is reached after 67 and 85 min, respectively^3,4^. Obesity and related abnormalities in the gall bladder mobility also increase the risk of gallstone formation^3,9,10^ more often in women compared to men. Women with a BMI > 45 kg/m^2^ have a sevenfold higher risk of gallstone formation as compared with those with a BMI < 24 kg/m^2^^11^. The relationship between the risk of cholesterol stone formation and the ratio of cholesterol to cholic acid (*ChA*) contained in bile should be considered while assessing the risk of gallstone formation^12^. In normal-weight subjects, the concentration of cholesterol in the bile pool may be up to nine times higher than that in the liver bile. Obesity causes increased secretion of the cholesterol and, as a consequence, its supersaturation in the gall bladder^3,7^. Prolonged presence of bile in the gall bladder, resulting from its impaired motility, is almost always observed in patients with cholesterol gallstone disease^13–15^. In this study, we considered the data obtained for men with BMI = 27.9 ± 3.2 kg/m^2^ and women with BMI = 29.1 ± 4.6 kg/m^2^. The total cholesterol in the blood serum of these men was estimated as 209.8 ± 44.4 mg/dL and in women as 208.6 + –29.2 mg/dL^2^. In addition, the data showed that obese men and women can exhibit a normal level of cholesterol. Because obesity, gallstone, and cholesterol circulation^16^ are correlated, we investigated the risk of gallstone formation in obese and normal-weight people with normal and elevated serum total cholesterol levels.

## Results

The analysis of the three-compartment model of cholesterol homeostasis enabled studying the effect of gall bladder motility on fluctuations in cholesterol concentration, as well as the assessment of the risk of cholesterol stone formation, in both normal-weight and obese people. In a normal-weight person, the fasting volume of gall bladder bile is 22 mL, of which 16 mL (ejection volume) is released into the duodenum during the contraction of the gall bladder, while in an obese person, the gall bladder releases 25 mL of bile (from fasting volume 33 mL to residual volume 8 mL). In both cases, the gall bladder releases bile at the same time, i.e., about 45 min. On the other hand, there are significant differences in gall bladder filling times between those with normal weight and those who are obese. By approximating the experimental filling curve to initial volume, we estimated the time of gall bladder refilling as 140 and 80 min for normal-weight and obese people, respectively^4^.

We began our research by fitting the solution for Eq. (3), which describes changes in the mass of *ChA*, to experimental data by adjusting *k*_g_, *M*_be_ and *s*_g_ parameters responsible for gall bladder filling rate, bile ejection, and storage ability, respectively.

Because the referred experimental data indicate the bile volume changes in the gall bladder, we converted them to mass changes, limiting only to the main bile component, i.e., *ChA*. We neglected the changes in the density of gall bladder bile (from 1.01 to 1.04 mg/mL), and furthermore, considered the average value, 1.025 mg/dL. In a normal-weight person, the gall bladder ejects 16 mL of bile, corresponding to the mass of 16.4 g, of which 9.14% is *ChA*, i.e., 1.499 g. On the other hand, in an obese person, the gall bladder ejects 25 mL, which corresponds to 2.342 g of *ChA*^17^.

The gall bladder motility of a normal-weight person can be modeled by Eq. (3) for values such as *k*_g_ = 2.5 × 10^−3^ (min^−1^), *s*_g_ = 4.7 × 10^−3^ (min^−1^), and *M*_be_ = 78 (mg min^−1^), while for an obese person the motility can be modeled with *k*_g_ = 6 × 10^−3^ (min^−1^), *s*_g_ = 5.4 × 10^−3^ (min^−1^), and *M*_be_ = 131 (mg min^−1^).

Since the parameters *m*_3total_, *k*, *w*, *m*_tis_, and *k*_b_ do not affect the gall bladder motility, we assumed their average values for both groups, i.e., *m*_3total_ = 6000 mg, *k* = 570 (mg^2^ min^−1^), *w* = 0.06, *m*_tis_ = 0.234 (mg min^−1^), and *k*_b_ = 0.57 (mg min^−1^).

For the established values of the aforementioned parameters, the total concentration of cholesterol in compartment II, *c*_2_ (peripheral blood), depends only on *a*_1_ and *a*_2_, the parameters that influence the amount of cholesterol transported with bile from and to the liver. The analysis of *c*_2_ changes resulting from the selection of *a*_1_ and *a*_2_ values obtained *c*_2_ = 180 mg/dL for normal-weight people, if the fraction *a*_1_/*a*_2_ was close to 2.5 × 10^−3^ (min^−1^), and *c*_2_ = 180 mg/dL if *a*_1_/*a*_2_ was 5.6 × 10^−3^ (min^−1^) for obese people.

**Figure 1:**
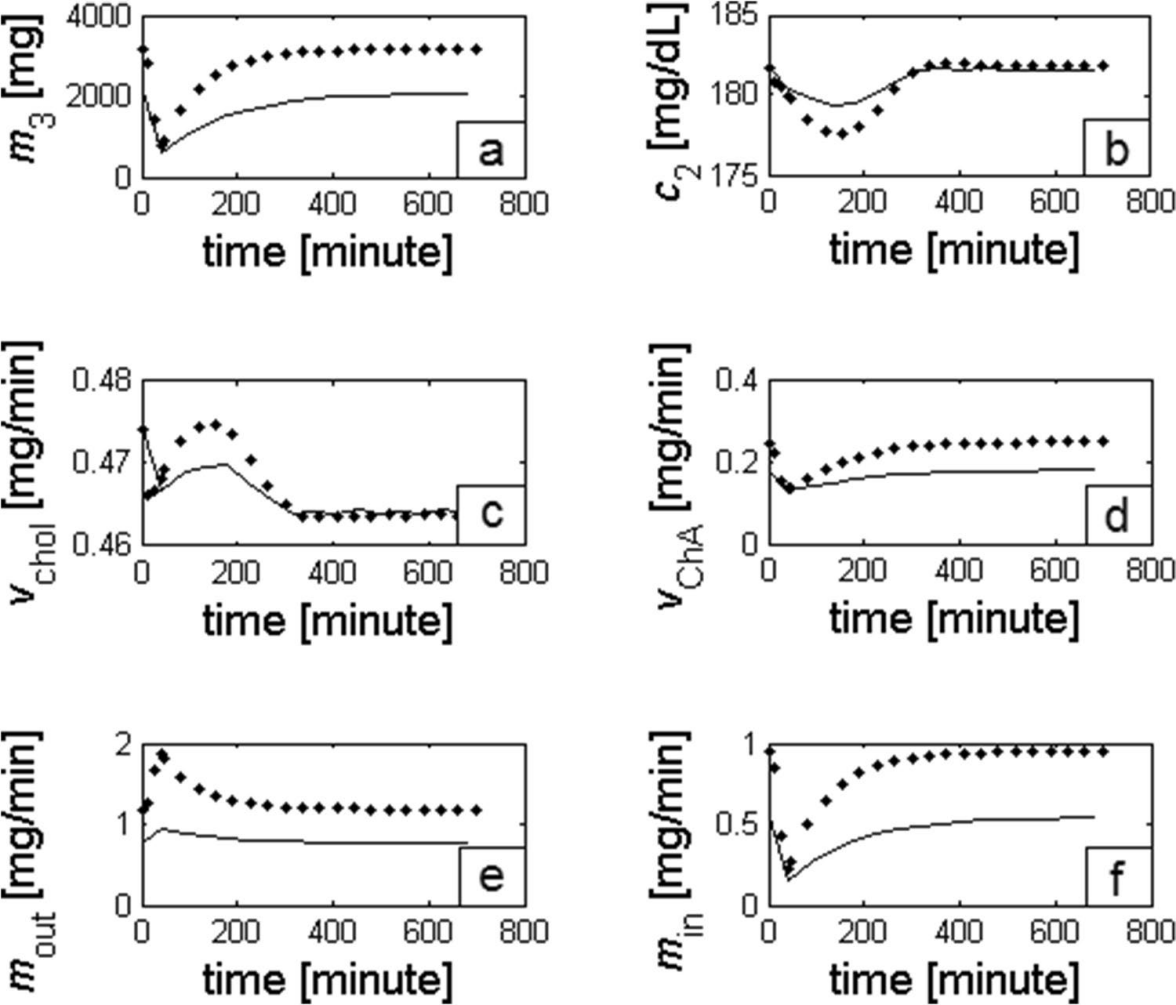
Results of the analysis for normal-weight (solid lines) and obese (dotted lines) persons, assuming that in both cases the total cholesterol concentration does not exceed 180 mg/dL. (**a**–**f**) Changes in the mass of *ChA* in the gall bladder, total cholesterol concentration *c*_2_ in the blood plasma, rate of de novo synthesis of cholesterol and *ChA*, the amount of cholesterol leaving the liver with bile, and the amount of cholesterol returning to the liver.

For individual values of parameters *a*_1_ and *a*_2_, the mass of cholesterol transported (within 12 h after the gall bladder contraction) from the liver (∑*m*_out_) and returned to the liver when the gall bladder is full, (∑*m*_in_), as well as the amount M^*^_in_ returned after bile ejection, were calculated. Integrating the solutions of *ChA*, *m*_out_, and *m*_in_ as a function of time in the range from 0 to 720 min, we obtained the total mass of *ChA* synthesized de novo as well as the amount of cholesterol leaving (∑*m*_out_) and returning (∑*m*_in_) with bile to the liver during 12 h after the gall bladder contraction (Table 1). In addition, based on equation (S9) (in the Supplementary Information), we calculated the mass (M^*^_in_) of cholesterol returning to the liver after earlier ejection from the gall bladder. Changes in the gall bladder content and the accompanying changes in *c*_2_ concentration, the rate of synthesis of cholesterol and *ChA*, and the amount of cholesterol transported with bile from and to the liver are shown in Fig. 1a–f.

**Table 1:** Parameters *a*_1_ and *a*_2_ allow maintaining the concentration of total cholesterol around 180 mg/dL. The other parameters represent the values observed in 12 h: ∑*m*_out_ is the amount of cholesterol carried by bile from the liver; ∑*m*_in_ is the amount of cholesterol returning to the liver which is unrelated to bile ejected from the gall bladder; *M*_in_^*^ is the mass of cholesterol returning to the liver along with bile after gall bladder contraction; and *ChA* is the mass of de novo-synthesized *ChA* from cholesterol particles.

*a*_1_ (min^−1^)	*a*_2_ (−)	∑*m*_out_ (mg/12 h)	∑*m*_in_ (mg/12 h)	*M*_in_* (mg/12 h)	*ChA* (mg/12 h)
**Normal-weight people**					
5.6 × 10^−4^	0.23	1831	1259	403	118
1.5 × 10^−4^	0.06	578	328	105	119
7.34 × 10^−4^	0.30	2362	1642	527	118
1.99 × 10^−4^	0.08	728	438	140	119
**Obese people**					
13 × 10^−4^	0.23	3153	2322	678	159
3.3 × 10^−4^	0.06	921	606	177	161
7.34 × 10^−4^	0.13	1850	1312	383	160
1.99 × 10^−4^	0.035	619	353	103	161

In addition, according to the experimental literature data, we observed an initial drop in the total cholesterol concentration (Fig. 1b); however, this effect was not associated with gall bladder motility^18^.

The greater ejection of bile as observed in obese versus lean individuals also leads to increased synthesis of cholesterol (Fig. 1c) as well *ChA* (Fig. 1d). Furthermore, the obtained results show that in obese people, the rate of cholesterol transport with bile from (Fig. 1e) and to the liver (Fig. 1f) is higher.

### Risk of cholesterol stone formation in people with normal weight and obese people with normal cholesterol levels

Table 1 shows the results for four selected examples of normal-weight and obese people with cholesterol levels not exceeding 180 mg/dL. In each of them, the risk of cholesterol stone formation can be assessed, which increases with an increase in the amount of cholesterol excreted in the liver into bile and decreases with an increase in the amount of de novo-synthesized *ChA* from cholesterol. From the phase diagram of the cholesterol/bile salts/phospholipid system^19^, a threshold value of the bile salts/cholesterol ratio of approximately 7.7 for the gall bladder bile of patients with stones could be determined. Here, for the analyzed cases, the total mass of cholic acid was assumed to be 6000 mg (corresponding to the bile salts). Considering the assessed limit value, the limit mass of cholesterol circulating with bile was estimated as 779 mg. Thus, we assumed that if the total mass of cholesterol carried by bile from the liver during a single gall bladder contraction (∑*m*_out_) was greater than this value, the risk of cholesterol stone formation increases. We also associated this risk in both groups to the threshold values of parameters *a*_1_ and *a*_2_.

In normal-weight people, we found a low risk for *a*_1_ with the minimum value of 1.99 × 10^−4^ min^−1^ and *a*_2_ = 0.08, which was close to the lower limit because the amount of cholesterol excreted in bile in 12 h was 729 mg. We determined a similar risk for the case with the minimum value of *a*_2_; however, for maintaining the cholesterol at 180 mg/dL, *a*_1_ = 1.5 × 10^−4^ min^−1^, i.e., below the estimated lower limit of variation, was selected. On the other hand, for *a*_1_ = 7.34 × 10^−4^ and 5.6 × 10^−4^ min^−1^, we found the highest risk of stone formation, because ∑*m*_out_ was found to be 2362 mg and ∑*m*_out_ was 1831 mg/12 h. In all the normal-weight people, the mass of newly synthesized *ChA* was similar, i.e., approximately 118 mg/12 h, and for maintaining *c*_2_ = 180 mg/dL, the ratio *a*_1_/*a*_2_ was required to be close to 2.5 × 10^−3^ (min^−1^).

In the obese group, normal cholesterol levels were maintained when the ratio *a*_1_/*a*_2_ was close to 5.6 × 10^−3^ min^−1^. For parameters *a*_1_ = 3.3 × 10^−4^ min^−1^ and *a*_2_ = 0.06, within the estimated limits (Supplementary Information), a slightly increased risk of stone formation was observed (∑*m*_out_ = 921 mg/12 h), which increased (∑*m*_out_ = 1850 mg/12 h) if *a*_1_ approached the upper limit of variation, i.e., *a*_1_ = 7.34 × 10^−4^ min^−1^ and *a*_2_ = 0.13. However, with the upper limit *of a*_2_, i.e., *a*_2_ = 0.23, *c*_2_ = 180 mg/dL was maintained when *a*_1_ significantly exceeded the upper limit (*a*_1_ = 13 × 10^−4^ min^−1^), but the risk of stone formation was significantly increased (∑*m*_out_ = 3153 mg/12 h). Low values of *a*_1_ (1.99 × 10^−4^ min^−1^, lower limit) and *a*_2_ (0.036, below the lower limit) are the most preferred because ∑*m*_out_ = 619 mg/12 h. In all the analyzed cases of obese people, we obtained a similar value of synthesized *ChA* of about 160 mg/12 h, which was almost 34% higher compared to that of normal-weight people.

In the second part of the study, we examined the effects of gradual weight gain resulting from improper diet and low physical activity. The analysis was started from the gall bladder motility of a normal-weight person, gradually transitioning toward the motility of an obese person, and finally, the amount of cholesterol secreted into the hepatic bile, de novo synthesis of *ChA*, the rate of *ChA* to cholesterol synthesis in the liver, and total cholesterol were determined.

The emptying and filling profile of the gall bladder depends on the amplitude of contraction of the muscles present in its walls, the rate of inflow of hepatic bile, and the ability to accumulate it. In the presented model, these processes are regulated by the parameters *M*_be_, *k*_g_, and *s*_g_. According to the previously determined values of *k*_g_, *M*_be_, and *s*_g_ for both normal-weight and obese people, we changed *k*_g_ from 2.5 × 10^−3^ to 6 × 10^−3^, *s*_g_ from 4.7 × 10^−3^ to 5.4 × 10^−3^ (min^−1^), and *M*_be_ from 78 to 131 (mg min^−1^). The change in *s*_g_ had a slight effect on the monitored parameters with which we associated the risk of stone formation. Since in obese people, the fasting volume of the gall bladder is higher than that of normal-weight people, while the emptying duration is similar between the groups, it is expected that the gall bladder may exhibit greater amplitude of contraction of its walls.

To avoid the negative mass of bile ejection, studies on the significance of *M*_be_ changes were carried out for *k*_g_ = 6 × 10^−3^ (min^−1^) and *s*_g_ = 5.4 × 10^−4^ (min^−1^), i.e., the parameters that are characteristic of obese people. Similar to the change in *s*_g_, an increase in *M*_be_ leads to slight changes in the parameters responsible for the process of stone formation. The greatest effects on changes in ∑*m*_in_, ∑*m*_out_, *ChA*, the ratio of de novo-synthesized *ChA* and cholesterol, and *c*_2_ are due to the decrease of the *k*_g_ parameter, as shown in Table 2, which also caused a gradual decrease in total cholesterol, while maintaining the parameters *a*_1_ and *a*_2_ as in a normal-weight person.

**Table 2:** Effect of gall bladder filling rate on the amount of cholesterol transported with bile, the amount of *ChA* synthesized during 12 h, the ratio of de novo-synthesized *ChA* to cholesterol, and total cholesterol in the blood plasma.

*k*_g_ (min^−1^)	∑*m*_in_ (mg/12 h)	∑*m*_out_ (mg/12 h)	*ChA* (mg/12 h)	*ChA*/cholesterol	*c*_2_ (mg/dL)
6.0 × 10^−4^	798.3	839.2	428.0	3.073	435
5.5 × 10^−4^	762.6	805.4	375.1	2.477	401
5.0 × 10^−4^	723.2	764.8	312.4	1.807	350
4.5 × 10^−4^	679.5	751.8	274.7	0.944	328
4.0 × 10^−4^	630.9	743.8	239.4	0.876	301
3.5 × 10^−4^	576.3	715.9	180.7	0.717	242
3.0 × 10^−4^	514.7	718.0	148.1	0.514	212
2.5 × 10^−4^	444.6	728.4	119.0	0.355	181

## Discussion

This study assessed the risk of cholesterol stone formation in normal-weight and obese people. Formation of cholesterol stones is more common among obese people compared to those with normal weight. These two groups are characterized by, inter alia, differences in gall bladder motility. In obese people, the gall bladder accumulates a larger volume of bile, which, through the contraction of its walls, is ejected into the duodenum in about 45 min. The duration of gall bladder ejection in obese people is similar to that of normal people, while the duration of gall bladder refilling is significantly shorter.

The emptying and filling of the gall bladder lead to fluctuations in the total cholesterol concentration in the peripheral blood. Cohn et al.^18^ reported the time profiles of changes in LDL, HDL and TG concentrations Based on Cohn’s et al. data and the Friedewald formula^20^, we estimated the changes in the total cholesterol and the initial decrease in it was consistent with our obtained results. Since in Cohn's experiment volunteers received a meal containing a control amount of cholesterol and fat, an increase was observed after the initial decrease in plasma cholesterol levels. In our in silico experiment, we assumed a cholesterol free diet containing fat what resulted in decrease and return to the initial level of the total cholesterol.

As already mentioned, in obese people, the gall bladder releases a larger amount of bile and is refilled faster and the correlated fluctuations in cholesterol concentration are greater compared to normal-weight people. Since the risk of cholesterol stone formation depends on the amount of cholesterol and *ChA* contained in the bladder bile^12^, we assumed that the amount of cholesterol leaving the liver with bile and the amount of de novo-synthesized *ChA* from cholesterol molecules may be related to the risk of stone formation. The more the amount of cholesterol in bile and the less the amount of synthesized *ChA*, the greater is this risk.

Comparison of the results obtained for both groups maintaining a normal cholesterol level showed that in both cases, the amount of cholesterol carried by bile (∑*m*_out_) can be lower (1000 mg/12 h) or higher (1800 mg/12 h), depending on the parameters *a*_1_ and *a*_2_. The estimated changes of these parameters were from 1.99 × 10^−4^ to 7.34 × 10^−4^ min^−1^ and from 0.06 to 0.23 for *a*_1_ and *a*_2_, respectively. When analyzing the risk of stone formation by considering the lower limit of *a*_1_, we observed ∑*m*_out_ was < 1000 mg/12 h in both normal-weight and obese people; however, in the latter group, *a*_2_ was estimated to have a significantly lower border value. If the upper limit of *a*_1_ was considered, which led to a significant increase in ∑*m*_out_, it was found that for the normal-weight group to maintain *c*_2_ = 180 mg/dL, *a*_2_ has to exceed the upper limit (*a*_2_ = 0.3).

Analogously, the *a*_2_ changes were analyzed. For a low value of *a*_2_ = 0.06 in both groups, the ∑*m*_out_ was below 1000 mg/12 h; however, for normal-weight people, *a*_1_ value should be below the estimated range (*a*_1_ = 1.5 × 10^−4^). If *a*_2_ = 0.23, i.e., a higher value, we observed a significant increase of ∑*m*_out_ in both groups; however, for obese people, it was much higher, and to maintain the normal *c*_2_ level, *a*_1_ has to be significantly increased to 13 × 10^−4^. Analyzing the importance of *a*_1_ and *a*_2_ for maintaining normal cholesterol levels, we found that for normal-weight people, the ratio *a*_1_/*a*_2_ should be close to 2.4 × 10^−3^ (min^−1^), while for obese people, the ratio should be almost twice higher, i.e., 5.6 × 10^−3^ (min^−1^).

Analysis of the data presented in Table 1 showed that the amount of newly synthesized *ChA* was in line with the literature data^21^. Since *ChA* is synthesized from cholesterol molecules, a 35% higher amount reduces the risk of stone formation in obese people.

### Changes in enterohepatic circulation accompanying weight gain

Analyzing the changes in the amount of cholesterol circulating with bile, the amount of de novo-synthesized *ChA* and cholesterol, as well as the total concentration of cholesterol associated with changes in gall bladder motility during weight gain, we found that the rate of bile flow into the gall bladder had the greatest impact on these values. The parameter determining the kinetics of this process is *k*_g_, which when increased in the gall bladder of a person—a characteristic of obesity—an increase in the amount of cholesterol in the enterohepatic circulation can be noticed, with a significant increase in total cholesterol concentration. In addition, we observed a significant increase in the ratio of de novo-synthesized *ChA* to cholesterol, mainly due to the increase in the synthesis of *ChA*, which is a natural defense against the formation of cholesterol stones. If the other parameters of the model retain their values, as seen in normal-weight people, then, based on the previously discussed results in obese people, maintenance of normal total cholesterol requires an increase in the *a*_1_/*a*_2_ ratio. As shown in Table 1, the increase in *a*_1_/*a*_2_ ratio in obese people is associated with an increased risk of cholesterol stone formation; however, when *a*_2_ is close to the lower limit (maintaining the *a*_1_/*a*_2_ value), the risk is low, which leads to the question of how to reduce the risk of cholesterol stone formation in obese people. Hypothetically, this can be achieved by lowering the *a*_1_ parameter, which determines the amount of cholesterol transported with bile from the liver, but this change is difficult to achieve from the current state of knowledge. However, by using a TG-lowering diet, we can reduce the risk of gallstone formation^11,22^ because it lowers the cholesterol absorption from the intestines in the form of chylomicrons (from the diet and from the membranes of dead enterocytes), owing to which the *a*_1_/*a*_2_ ratio increases toward maintaining normal total cholesterol. The obtained results show the benefit of the reduction of enterohepatic circulation in obese people, which can be achieved in different ways: inhibition of *ChA* synthesis, increasing the duration of gall bladder emptying, reducing the amount of bile released during gall bladder contraction, and increasing its elimination with feces. The rate of *ChA* synthesis can be reduced by inhibiting the enzymes cholesterol 7α hydroxylase and sterol 27-hydroxylase (CYP27A1) that initiate the complex process (involving 17 enzymes). Activity of 7α-hydroxylase in obese people was found to be almost twice as high as that in the control group^23^. However, since a significant reduction in *ChA* synthesis can lead to an unfavorable cholesterol/*ChA* ratio, the simultaneous inhibition of cholesterol synthesis seems reasonable. The gall bladder emptying duration can be increased by consuming meals containing a small amount of pure fat^11,24^ as well as through exercise that may affect gall bladder motility^8,11^ by the stimulation of vagus nerve, while the amount of bile ejection can be reduced by using inhibitors, e.g., loxiglumide or MK-329^15,25^, of receptors for cholecystokinin (CCK)^26^. Consequently, obese people should avoid taking choleretic agents and a diet that increases gall bladder motility. Increased removal of cholesterol, along with bile, from the intestines can be achieved using anion-exchange resin; however, it increases the release of CCK^15^.

## Methods

Since enterohepatic circulation affects the cholesterol level^27,28^ with the gall bladder playing an important role, we extended our model of cholesterol homeostasis^29^ with a third compartment, leading to the analysis of the risk of gallstone formation associated with the amount of cholesterol circulating with bile. The study of the behavior of the three-component system, consisting of cholesterol, bile salts, and phospholipids in the gall bladder bile of normal subjects and patients with gallstones resulted in a line of maximum cholesterol solubility^30^. This curve, separating the liquid phase and the phase containing crystals, is nearly flat with the percent change of cholesterol ranging from 5 to 10%. For bile containing less than 40% bile salts, cholesterol does not form a soluble phase; however, in people with gallstones, bile salts account for 55–95%. By analyzing the phase diagram, it can be concluded that a relatively small increase in the amount of cholesterol in the gall bladder bile, to above 10%, increases the probability of stone formation regardless of the amount of phospholipids. Lowering the bile salt content also promotes the formation of cholesterol stones. However, the presented model does not consider lipids in bile composition; hence, we limited the risk assessment of cholesterol stone formation to the analysis of the amount of cholesterol circulating with bile and the de novo-synthesized *ChA*, which is produced from cholesterol in the liver.

Because stone formation is prolonged, we assumed that the risk of stone formation is higher with more amount of cholesterol circulating with bile and less number of new *ChA* molecules formed.

**Figure 2:**
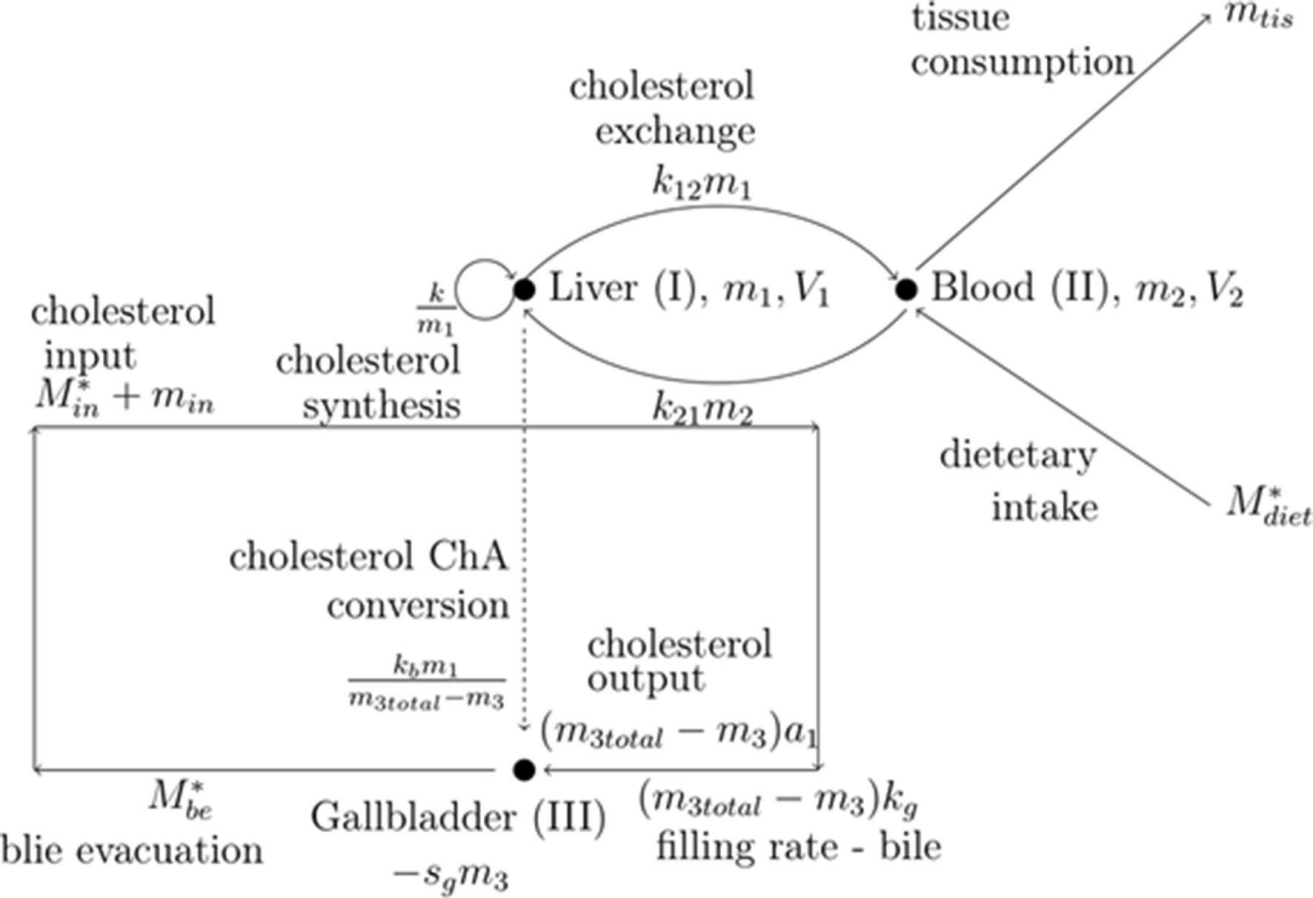
Schematic representation of the three-compartment model of cholesterol homeostasis in the human body. The three compartments refer to the following: *m*_1_, *V*_1_—the mass of cholesterol in the volume of blood plasma in the liver (I); *m*_2_, *V*_2_—the mass of cholesterol in the volume of peripheral blood plasma (II); and *m*_3_, *V*_3_—the mass of *ChA* in the volume of the gall bladder (III). Dashed line represents the conversion of cholesterol into *ChA* (one cholesterol molecule is converted into one *ChA* molecule). Therefore, *k*_b_*m*_1_/(*m*_3total_ − *m*_3_) represents the outflow of cholesterol from compartment I and the entry of *ChA* into compartment III.

The presented three-compartment model of cholesterol homeostasis is based on the scheme of cholesterol circulation (Fig. 2), which includes: cholesterol and *ChA* (the main bile component synthesized from cholesterol) de novo synthesis, cholesterol tissue consumption, cholesterol exchange between the liver and peripheral blood, cholesterol circulation with bile, cholesterol loss with the feces, and dietary cholesterol. The rate of cholesterol synthesis depends on the amount of cholesterol present in the blood flowing through the liver (compartment I). The lower the concentration of cholesterol in the liver cells, the faster the rate of its synthesis^31^, and it can be expressed as the quotient of *k*/*m*_1_, where *k* is the kinetic constant and *m*_1_ is the mass of cholesterol in compartment I. As the volumes of the considered compartments are not equal, we do not include the concentrations but the masses. However, if we want to refer to the lipid profile tests, we must divide the cholesterol mass by the blood plasma volume of the given compartment.

De novo synthesis of cholic acid (*ChA*) also takes place in the liver, and the rate of this process increases with the increasing amount of cholesterol and decreasing amount of *ChA* returned to the liver. This process can be described by the expression *k*_b_*m*_1_/(*m*_3total_ − *m*_3_), where *k*_b_ is the kinetic constant, and *m*_3total_ and *m*_3_ are the total mass of *ChA* and the current mass of *ChA* in the gall bladder (compartment III), respectively. Here, we simplified the bile composition only to include the main component, i.e., *ChA*, and changes in *ChA* accumulation in the gall bladder (compartment III) depend on the gall bladder filling rate, expressed by bile inflow—(*m*_3total_ − *m*_3_)*k*_g_, *ChA* de novo synthesis from cholesterol—*k*_b_*m*_1_/(*m*_3total_ − *m*_3_) (we neglected the relatively minor difference in the molar mass of cholesterol and *ChA*), gall bladder emptying rate M^*^_be_, and gall bladder’s bile-accumulating ability *s*_g_*m*_3_, where *k*_g_ (min^−1^) and *s*_g_ (min^−1^) represent, respectively, the kinetic constant of gall bladder filling and the gallbladder’s ability to accumulate bile. If we neglect the amount of *ChA* in the bile ducts and portal vein, then the expression (*m*_3total_ − *m*_3_) corresponds to the *ChA* mass in the liver.

As cholesterol is needed by every cell and its synthesis takes place mainly in liver cells, the model should consider this demand represented by a time-averaged *m*_tis_ value. Therefore, a separate compartment for peripheral blood should be introduced accounting for this process (compartment II). In addition, this compartment should necessarily describe the reverse transport of cholesterol from the peripheral blood to the liver (e.g., cholesterol recovered from the membranes of dead cells during the process of apoptosis). The rate of cholesterol exchange between compartments I and II is described by the expressions *k*_12_*m*_1_ and *k*_21_*m*_2_, where *k*_12_ and *k*_21_ are the effective kinetic constants of a multistep process and *m*_1_ and *m*_2_ refer to cholesterol mass in compartments I and II.

To consider the enterohepatic circulation of cholesterol carried by bile, we introduced a third compartment—the gall bladder. The rate of change of the *ChA* mass present in compartment III has been described above. As circulating bile always contains a certain amount of cholesterol, the parameter *a*_1_ was introduced, defining its content in the incoming bile, which can be expressed as (*m*_3total_ − *m*_3_)*a*_1_. As mentioned earlier, de novo synthesis of *ChA* from cholesterol molecules takes place in the liver and if we neglect the relatively small difference in the molecular masses of both molecules, the same expression can describe a decrease in cholesterol mass in compartment I and an increase in *ChA* mass in compartment III. Reverse cotransport of cholesterol with the bile to the liver can be divided into two components: constant *m*_in_—when the gallbladder is full and varying in time M^*^_in_—after ejection from the gall bladder. The mass of *ChA* returning to the liver can be written as (*m*_3total_ − *m*_3_^~^)*k*_g_(1 − *w*), where *m*_3_^~^ is the maximal mass of *ChA* accumulated in the gall bladder and *w* refers to *ChA* loss with fecal masses. To consider the amount of cholesterol transported by *ChA* to the liver, we introduced the parameter *a*_2_. Finally, *m*_in_ = (*m*_3total_ − *m*_3_^~^)*k*_g_(1 − *w*)*a*_2_. The same value of parameter *a*_2_ is used in the description of cholesterol returning to the liver after the gall bladder emptying (for details, see Supplementary Eqs. S9 and S10 in the Supplementary Information). The range of model parameter variability is presented in the Supplementary Information.

Because dietary cholesterol can be easily eliminated by a vegetarian diet, we rejected this factor through an analysis of the risk of gallstone formation in both normal-weight and obese people with an elevated cholesterol level. Thus, the three-compartment model is represented by Eqs. (1–3).

1 $$ \frac{{dm_{1} }}{dt} = \frac{k}{{m_{1} }} + k_{21} m_{2} - k_{12} m_{1} - \left( {m_{{{\text{3total}}}} - m_{3} } \right)a_{1} - k_{b} \frac{{m_{1} }}{{m_{{{\text{3total}}}} - m_{3} }} + m_{{{\text{in}}}} + M_{{{\text{in}}}}^{*} , $$

2 $$ \frac{{dm_{2} }}{dt} = - k_{21} m_{2} + k_{12} m_{1} - m_{{{\text{tis}}}} + M_{{{\text{diet}}}}^{*} , $$

3 $$ \frac{{dm_{3} }}{dt} = - M_{be}^{*} - s_{g} m_{3} + \left( {m_{{{\text{3total}}}} - m_{3} + k_{b} \frac{{m_{1} }}{{m_{{{\text{3total}}}} - m_{3} }}} \right)k_{g} . $$

Equations (1) and (2) describe the rate of changes in the mass of cholesterol occurring in compartment I (the blood plasma flowing through the liver) and in compartment II (in the peripheral blood plasma). The third equation describes the rate of change in the mass of *ChA* occurring in the gall bladder.

All parameters describing the aforementioned processes occurring in varying time intervals are marked with asterisks. As we have shown earlier^29^, their dependence on time can take the form *A* sin^2^(*ωt*), where *A* is the amplitude of a given process (expressed in mg min^−1^) and *ω* is the angular pulsation, which is bound with *t*_b_ and *t*_e_, i.e., the start and end times of a given process shown in Eq. (4):

4 $$ \omega = \frac{\pi }{{t_{e} - t}}_{b} . $$

The rate of changes in model parameters was estimated based on the knowledge of the physiology of the studied processes (details are given in the Supplementary Information).

Based on Eq. (3), we derived Eq. (5) for fasting mass of *ChA*, *m*_3_^~^, corresponding to the fasting volume of gall bladder (details are given in the Supplementary Information):

5 $$ m_{3}^{\sim } = \frac{{m_{{3{\text{total}}}} \left( {s_{g} + 2k_{g} } \right) - \sqrt { - 4k_{b} k_{g}^{2} m_{1} - 4k_{b} k_{g} m_{1} s_{g} + m_{{3{\text{total}}}}^{2} s_{g}^{2} } }}{{2\left( {s_{g} + k_{g} } \right)}}. $$

According to Eq. (5), the maximum *ChA* mass accumulated in the gall bladder depends on: *m*_3total_, *k*_g_, *s*_g_, and *m*_1_, where *m*_1_ is the mass of cholesterol contained in the blood flowing through the liver.

To solve Eqs. (1–3), we used the Runge–Kutta method, coded in MATLAB (version 2007b, solver ode45). We assumed equal concentrations of cholesterol in the blood flowing through the liver and in the peripheral blood as the initial values. Assuming, as previously^29^, in a 70 kg man, with 6.5 and 23.5 dL of blood plasma in the liver and in the peripheral vessels, we obtained initial masses for compartments I and II as the product of cholesterol concentration and the corresponding plasma volume. From Supplementary Eq. S5  (in the Supplementary Information) derived for a completely filled gall bladder, we calculated the initial mass *m*_3_ (of *ChA*) for compartment III.

## Supplementary Information

Supplementary Information.
