# Supplementary material for: A computer study of the risk of cholesterol gallstone associated with obesity and normal weight
Source: Sci Rep. 2021 Apr 23;11:8868. doi: 10.1038/s41598-021-88249-w (PMC8065120; doi:10.1038/s41598-021-88249-w)
Supplement: Supplementary file 1 — Supplementary Information. [file 41598_2021_88249_MOESM1_ESM.rtf]

Supplementary Information
A computer study of the risk of cholesterol gallstone associated with obesity and normal weight

Krystian Kubica1*, Joanna Balbus2
*corresponding author: krystian.kubica@pwr.edu.pl, orcid.org/0000-0002-8814-0715
1Faculty of Fundamental Problems of Technology, Department of Biomedical Engineering, Wroclaw University of Science and Technology, 50-370, Wroclaw, Poland
2Department of Pure and Applied Mathematics, Wroclaw University of Science and Technology, 50-372, Wroclaw, Poland


The range of changes of model parameters has been estimated on the basis of knowledge of the physiology of the studied processes. It was assumed that at time ô0, the gall bladder was completely filled with bile and that cholesterol concentrations in compartment I (peripheral blood plasma) and in compartment II (liver blood plasma) were the same and equal to 180 mg dL-1. Thus, for 6.5 dL of plasma in the liver and 23.5 dL of peripheral blood plasma, we obtained the initial mass of cholesterol in compartments I and II: m1(ô0)=1170 mg, and m2(ô0)=4230 mg, respectively.

We consider an equation describing the rate of change in the mass of the cholic acid in the gall bladder
. 		 (S1)
Equation (S1) for  (no gall bladder contraction) has a two stationary points
 ,			(S2)
and
 ,			(S3)

Denote for  
 		.  (S4)
For  sg=4.7*10-3 min-1, m3total=6000 mg, kg= 2.5*10-3 min-1, m1=1170 mg, kb=0.57 mgmin-1
 (it means that the point   is locally unstable stationary point)
and  (it means that the point  is locally stable stationary point).
Now we show that the point  is asymptotically stable stationary point.
The solution of the equation (S1) take a form 
    				(S5)
where 	 , ,
and m3(0) means a value of function m3 at time 0
If  	then	 . 
We observe that the right side of the Equation (S5) goes to 0 if t goes to infinity. Then the left side of Equation (S5) goes to 0 if  goes to  as t goes to infinity. It means that the point  is asymptotically stable stationary point and it means that equation  (S3) determines the maximum amount of ChA accumulated in the gall bladder.

Range of changes of model parameters:
1.	k
The rate of de novo synthesis of cholesterol is expressed by the term k/m1, and it varies from 0.324 to 0.625 mg min-1 . Yasiro, M., Muso, E.,  Matsushima, M., Nagura, R., Sawanishi, K. & Sasayama, S. Two- 
    compartment model of cholesterol kinetics for establishment of treatment strategy of LDL  
    apheresis in nephrotic hypercholesterolemia. Blood Purificat. 12, 317–326, (1994).   
    https://doi:10.1159/000170180.. Thus, for the assumed mass m1=1202 mg, k takes values ranging from 389 to 751 mg2 min-1 .

2.	k12, k21, mtis
Values of parameters k12 and k21, which describe the rate of cholesterol exchange between compartments I and II, are the same as those in our previous work . Hrydziuszko, O., Wrona, A., Balbus, J. & Kubica, K. Mathematical two-compartment model of   
    human cholesterol transport in aplication to high blood cholesterol diagnosis and Treatment.        
     Elecrtronic Notes In Theoretical Computer Science. 306, 19-30, (2014). 
  https://doi: 10.1016/j.entcs.2014.06.012.,. Hrydziuszko, O., Balbus, J., ¯ulpo, M., Wrona, A. & Kubica, K. Mathematical analyses of two-  
    compartment model of human cholesterol circulatory transport in application to high blood 
    cholesterol prevention, diagnosis and treatment. Theor. Comput. Sci. 608, 98-107, (2015).  
    https://dx.doi.org/10.1016/j.tcs.2015.07.057., i.e. k12=3.58 min−1 and k21=1.0 min−1. The parameter k21 was normalized to one, while k12, as mentioned in Section 2.1, refers to the ratio of LDL/HDL=3.5 . Berg, J. M., Tymoczko, J. L. & Stryer, L. Biochemistry, 6th ed. (New York: WH. Freeman, 2006)..
Because the variation in tissue demands on cholesterol has been inadequately explored, we used the average value expressed in mg min-1, i.e. mtis=0.243 mg min-1 . Sabine, J. R. Cholesterol. (New York, Mercel Dekker, 1977). To study the impact of this parameter on the total cholesterol c2, we examined the model's response to ±50% variation of it.
3.	m3total
The parameter m3total takes the value that refers to the sum of mass of ChA in the liver and gall bladder. Because our model did not include ChA contained in the bile ducts, intestines, and portal vein, we assume that the m3total value will be lower than the total ChA, i.e. from 4000 to 8000 mg . Guyton, A. C. & Hall, J. E. Textbook of Medicinal Physiology, 13th ed. Philadelphia: Elsevier; 
    (2016). It was initially assumed that m3total=6000 mg.
4.	kb
The parameter kb determines the speed of synthesis of ChA, which should be in the range from 0.14 to 0.41 mg min-1 . Chiang, J. Y. L. Bile acids: regulation of synthesis. J. Lipid Res. 50, 1955-1966, (2009).. In our model, the speed is represented by the term kbm1/(m3total−m3). For the already assumed m1(ô0)=1202 mg and because the mass of ChA contained in the gall bladder is equal to half of m3total, kb varies between 0.233 and 1.364 mg min-1 for m3total=4000 and 8000 mg, respectively.

5.	sg, kg
Estimation of the parameters sg and kg was based on the steady-state calculations. Namely, for a fixed mass of cholesterol in compartment I (m1'), the stationary solution dm3/dt=0 of Equation (S1), for a filled gall bladder,  gives Equation (S3), which refers to the maximum mass m3~  of ChA, collected in the gall bladder.
      
Because the expression under the root in Equation (S3) cannot be negative, parameters sg, kg, kb, and m3total have to meet condition (S6):

				(S6)
					

The liver secretes 600–1000 cm3 of bile per day (Guyton and Hall 2016). In 1000 cm3 of bile, there is approximately 7 g of ChA. This means that in 1 minute, 2.91–4.86 mg of this acid leaves the liver and passes from the common hepatic duct into the gall bladder via the cystic duct. In our model, this process is expressed by the term (m3total−m3)kg. For the considered range of m3total (4000–8000 mg) and the assumption that the gall bladder stores half of it, kg changes between 0.73×10^−3^ and 2.43×10−3 min−1.
Next, for limit values kg=0.727×10−3 min−1, kb=0.233 mg min-1, m3total=4000 mg, and m1'=m1(ô0)=1202 mg, based on inequality (A6) we obtained  min−1. For kg=2.43×10−3 min−1, kb=1.364 mg min-1, and m3total=8000 mg, sg > 0.247x10-4 min-1.

6.	w
The loss of bile acids with faeces can be determined by setting the w value. Approximately 6% of the circulating bile acids are removed with faeces 6.

7.	a1
Cholesterol egress from compartment I depends on two terms: synthesis of ChA from cholesterol (kbm1/(m3total−m3)) and cholesterol cotransport with the circulating ChA (m3total−m3)a1, where a1 is the kinetic constant. The sum of these expressions determines the average cholesterol egress from compartment I and is equal to 1.2 [mg min-1]  3. The content of m3 in the gall bladder varies from 25% (for the emptied gall bladder) of the maximum mass contained therein, i.e. to m3~ (for the maximum filled gall bladder) . Kossena, G. A., Charman, W. N. &Wilson, C. G. et al.  Low dose lipid formulations: effects on 
    gastric emptying and biliary secretion. Pharm. Res. 24(11), 2084-96, (2007). . For limit values of m3total (4000–8000 mg) and kb (0.233–1.364 mg min-1), the parameter a1 calculated from Equation (S7) varies between 1.99×10^−4^ and 7.32×10−4 min−1 for the empty and filled gall bladder. 

.				(S7)

8.	a2
Cholesterol entry with bile is divided into the constant component min and the variable component M*in. The constant value of min can be expressed by the amount of ChA flow when the gall bladder is full, that is:

	.				(S8)

To estimate the average value of the variable component Min*, we have to estimate the value of Mbe first. We can estimate it for ô1=30 min, m3~=0.5m3total, and according to Equation (S8), Mbe varies between 100 and 200 mg min-1 for m3total=4000 and 8000 mg, respectively. Now, using Equation (11), we obtain Min=11.88 mg min-1 for Mbe=200 mg min-1, ô1=30 min, ô2=60 min, ô3=565 min, and w=0.06. For the lower limit of parameters: Mbe=100 mg min-1, ô2=145 min, ô3=320 min, we obtain Min=13.95 mg min-1.
Thus, the amount of cholesterol returning to the liver with a speed Min* in the period ô2–ô3, carried by bile that had previously left the gall bladder in the period ô0–ô1 according to Equation 6, can be calculated as shown in Equation (S9):
.				(S9)

For Min=(11.88–13.95) mg min-1, Mbe=(100–200) mg min-1, ô2=(60–145) min, ô3=(360–565) min, the total cholesterol returning with the bile to the liver is (1499.6–2999.7)a2. In our previous work, the average mass of cholesterol returning with the bile to the liver per minute was estimated to be 0.8 mg min-1. Here, the average mass consists of two components: the average constant component and the average variable component, which can be written in the form of Equation S10:

,			(S10)

where B=(1499.6–2999.7).
For already estimated ranges of variation of parameters kg, m3total, m3~, parameters known from physiology, that is, w=6%, ô2=(60–145) min, and ô3=(360–565) min, and calculated Min=(11.88–13.99) mg min-1, we have a2 in the range 0.060–0.232.

References
